# Fragment screening reveals salicylic hydroxamic acid as an inhibitor of *Trypanosoma brucei* GPI GlcNAc-PI de-*N*-acetylase

**DOI:** 10.1016/j.carres.2013.12.016

**Published:** 2014-03-31

**Authors:** Michael D. Urbaniak, Amy S. Capes, Arthur Crossman, Sandra O’Neill, Stephen Thompson, Ian H. Gilbert, Michael A.J. Ferguson

**Affiliations:** Division of Biological Chemistry and Drug Discovery, College of Life Sciences, University of Dundee, Dow Street, Dundee DD1 5EH, UK

**Keywords:** GPI, *Trypanosoma brucei*, Hydroxamic acid, Inhibitor, *N*-Deacetylase

## Abstract

The zinc-metalloenzyme GlcNAc-PI de-*N*-acetylase is essential for the biosynthesis of mature GPI anchors and has been genetically validated in the bloodstream form of *Trypanosoma brucei*, which causes African sleeping sickness. We screened a focused library of zinc-binding fragments and identified salicylic hydroxamic acid as a GlcNAc-PI de-*N*-acetylase inhibitor with high ligand efficiency. This is the first small molecule inhibitor reported for the trypanosome GPI pathway. Investigating the structure activity relationship revealed that hydroxamic acid and 2-OH are essential for potency, and that substitution is tolerated at the 4- and 5-positions.

## Highlights

• First non-substrate analogue inhibitor of the trypanosome GPI pathway.
• Active against recombinant enzyme and cell-free system.
• Low molecular weight and good ligand efficiency.

## Graphical abstract

**Figure:**
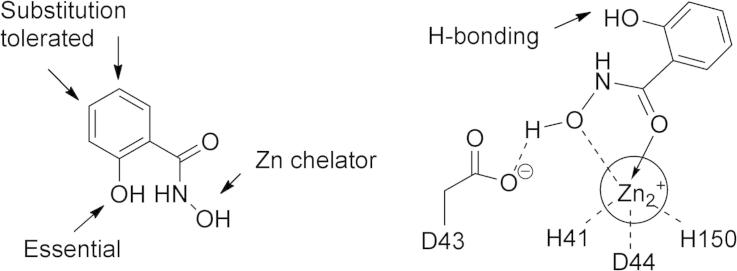

## 1 Introduction

*Trypanosoma brucei* is a protozoan parasite that is transmitted by the bite of an infected tsetse fly and causes the fatal African sleeping sickness in humans and the related wasting disease Nagana in cattle. Current treatments are expensive, toxic, and difficult to administer, leaving an urgent need for improved therapeutic agents. The parasite is able to evade the host immune response by virtue of a dense surface coat composed of 5 × 10^6^ variant surface glycoprotein (VSG) dimers that prevent lysis by the innate immune response. The coat also undergoes antigenic variation to evade the adaptive immune response.^1^ The VSG dimer is attached to the plasma membrane by a glycosylphosphatidylinositol (GPI) anchor, and GPI anchor biosynthesis has been genetically and chemically validated as essential for the survival of the clinically relevant bloodstream form of the parasite.^2–5^

**Figure 1:**
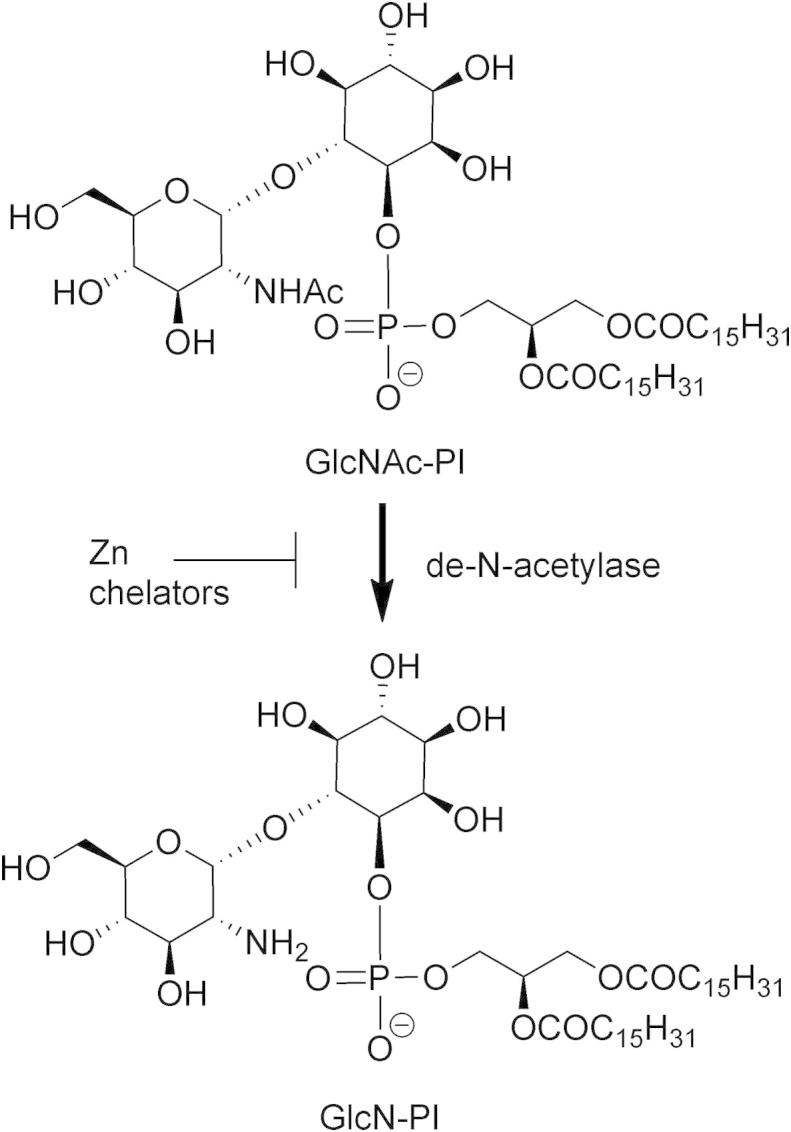
GlcNAc-PI de-*N*-acetylase catalyses the second step in the GPI biosynthetic pathway. The zinc-dependent metalloenzyme is inhibited by zinc chelators.

The GPI biosynthetic pathway has been extensively studied in both *T. brucei* and mammalian systems, highlighting differences in both the order of assembly and substrate specificity that can be exploited to produce species-specific inhibitors. The GlcNAc-PI de-*N*-acetylase is a zinc metalloenzyme that catalyses the second step in biosynthesis of GPI (Fig. 1),^6^ and has been genetically validated as essential in bloodstream form *T. brucei*.^3^ The use of synthetic substrate analogues has revealed that the human enzyme is more fastidious than the trypanosome enzyme, enabling the design of species-specific substrate-based inhibitors.^7–9^ The substrate analogue approach has shown that the phosphate, 2′-NHAc and 3′-OH of the substrate GlcNAc-PI are critical for recognition by the *T. brucei* GlcNAc-PI de-*N*-acetylase.^10^ In contrast, the diacylglycerol is not directly recognised and may be efficiently replaced with an octadecyl chain.^9,11^ Recently, we have shown that substrate-based inhibitors containing the zinc binding moieties hydroxamic acid or carboxylic acid can act as inhibitors of the *T. brucei* de-*N*-acetylase.^12,13^ These substrate-based inhibitors do not possess drug-like physiochemical properties and contain too much stereochemical complexity for tractable synthesis. Here, we report our efforts to identify alternative scaffolds for GlcNAc-PI de-*N*-acetylase inhibitors that resulted in the identification of salicylic hydroxamic acid as an inhibitor with high ligand efficiency.

## 2 Materials and methods

### 2.1 Materials

The synthesis of d-glucosamine-α-(1-6)-d-myo-inositol-1-octadecyl phosphate (GlcN-I*P*C_18_) has been described previously.^14^ The corresponding *N*-acetyl derivate (GlcNAc-I*P*C_18_) was prepared by treatment with acetic anhydride, and the concentration of stock solutions determined by measurement of the inositol content by selected ion-monitoring GC–MS.^13^ Bloodstream form *Trypanosome brucei* (variant MITat1.4) was isolated and membranes (cell-free system) prepared as described previously and stored at −80 °C.^15^ Recombinant GST-tagged *T. brucei* de-*N*-acetylase (GST-*Tb*GPI12) was expressed and purified as described previously and stored at −80 °C.^13^

### 2.2 Mass spectrometry based activity assays

Inhibition assays were performed in 100 μL final volume, with 1% v/v DMSO with or without inhibitor. Recombinant GST-*Tb*GPI12 (10 μg per assay) or trypanosome cell-free system (2.5 × 10^6^ cell equivalents per assay) in incorporation buffer (25 mM Tris pH 8.0, 50 mM KCl, 5 mM MnCl_2_) was added to wells containing 500 pmol GlcNAc-I*P*C_18_ with or without the inhibitor and incubated at 37 °C for 1 h. The reactions were quenched by the addition of 200 μL of 5% propan-1-ol, 5 mM NH_4_OAc, and the glycolipids were bound to C_18_ resin (50 mg Isolute cartridge), washed three times with 200 μL 5% propan-1-ol, 5 mM NH_4_OAc and eluted with 100 μL 40% propan-1-ol, 5 mM NH_4_OAc. Enriched glycolipids were analysed by liquid chromatography coupled to an electrospray tandem mass spectrometer (LC–MS/MS). Samples (40 μL) were injected on to a 10 × 1 mm C_18_ column (ACE, 5 μM) and eluted using a binary gradient of 5–80% propan-1-ol in 5 mM NH_4_OAc (Dionex Ultimate 3000). The gradient consisted of 2 min 0% B, 2–4 min 0–100% B, 4–8 min 100%, 8–9 min 100–0% B, 9–10 min 0% B where buffer A consisted of 5% propan-1-ol, 5 mM NH_4_OAc and buffer B 80% propan-1-ol, 5 mM NH_4_OAc. The glycolipids were analysed on an electrospray triple quadrapole mass spectrometer (Micromass Quattro Ultima) in multiple reaction monitoring mode. The turnover of the substrate GlcNAc-I*P*C_18_ (*m*/*z* 715 > 223) to GlcN-I*P*C_18_ (*m*/*z* 672 > 223) was used to calculate the percentage of substrate conversion to product in a given sample.^6^ Inhibitor IC_50_ values were calculated using a four-parameter fit of eight-point potency curves derived from three independent experiments, and are quoted with standard deviation.

### 2.3 Trypanosome cell-free system assay

Trypanosome cell-free system assays, where the formation of GPI precursors is monitored by following the incorporation of [^3^H]-mannose were analysed using high-performance liquid chromatography and fluorography as described previously.^13^

### 2.4 Compound synthesis

#### 2.4.1 Synthesis of 5-((*tert*-butoxycarbonyl)amino)-2-hydroxybenzoic acid (**9**)

5-Aminosalicylic acid **8** (6.89 g, 45.0 mmol) was suspended in water (60 mL), and TEA (12.4 mL, 90.0 mmol) was added. A solution of Boc_2_O (10.8 g, 90.0 mmol) in dioxane (120 mL) was added, and the reaction was stirred at room temperature overnight. The solvent was removed, and the solid residue suspended in water (20 mL). HCl (3 M) was added until the greyish pink precipitate stopped forming. The precipitate was filtered off and washed with water. The precipitate was dissolved in boiling acetone and hot filtered before being recrystallized twice from acetone to afford the product (8.61 g, 76%) as a white powder, mp 278 °C. ^1^H NMR (500 MHz, MeOD) *δ* 7.93 (1H, s), 7.40 (1H, d, *J* = 7.6 Hz), 6.85 (1H, d, *J* = 8.9 Hz), 1.51 (9H, s); ^13^C NMR (125 MHz, MeOD) *δ* 173.3, 159.0, 155.7, 132.1, 128.7, 121.8, 118.2, 113.5, 80.8, 28.7; HRMS, calcd mass for C_12_H_16_NO_5_^+^ [M+H^+^]: 254.1023. Found: 254.1027 (−1.4 ppm).

#### 2.4.2 Synthesis of *tert*-butyl (3-((benzyloxy)carbamoyl)-4-hydroxyphenyl)carbamate (**10**)

*O*-Benzylhydroxylamine hydrochloride (3.54 g, 22.2 mmol) was suspended in CHCl_3_ (85 mL) and cooled to 0 °C, and then TEA (3.21 mL, 23.1 mmol) was added. A solution of compound **9** (5.62 g, 22.2 mmol) in THF (140 mL), DMAP (169 mg, 1.12 mmol) and DCC (5.06 g, 24.4 mmol) was added, and the reaction was stirred for 16 h. The solvent was removed and the solids were resuspended in EtOAc, filtered, and then washed with HCl (1 M, 50 mL), water (50 mL), ammonia (1 M, 2 × 50 mL) and filtered through cotton wool. The solvent was removed and the crude was dried on to silica then purified by column chromatography (EtOAc/hexane, 0:100 to 1:1) to afford the product (3.63 g, 46%) as a pale yellow powder, mp 102–103 °C. ^1^H NMR (500 MHz, CDCl_3_) *δ* 11.33 (1H, br s), 9.42 (1H, br s), 7.63 (1H, br s), 7.44–7.41 (2H, m), 7.39–7.34 (3H, m), 7.13 (1H, dd, *J* = 8.9, 2.4 Hz), 6.90 (1H, d, *J* = 8.9 Hz), 6.45 (1H, br s), 4.97 (2H, s), 1.49 (9H, s); ^13^C NMR (125 MHz, CDCl_3_) *δ* 157.1, 153.3, 134.9, 129.6, 129.3, 128.9, 128.7, 128.7, 126.1, 118.8, 116.0, 112.1, 80.8, 78.6, 28.3; LRMS (ES^+^), *m*/*z* 359.2 [M+H^+^]; HRMS, calcd mass for C_19_H_23_N_2_O_5_^+^ [M+H^+^]: 359.1601. Found: 359.1591 (2.8 ppm).

#### 2.4.3 Synthesis of 5-amino-*N*-(benzyloxy)-2-hydroxybenzamide TFA salt (**11**)

Compound **10** (1.99 g, 5.56 mmol) was dissolved in wet TFA (5.3 mL), and the mixture was stirred for 1 h. The TFA was removed in vacuo; the residue was suspended in Et_2_O and filtered to afford the product (1.76 g, 85%) as a tan powder, mp 169–170 °C. ^1^H NMR (500 MHz, DMSO-*d*_6_) *δ* 11.48 (1H, br s), 9.96 (2H, br s), 7.64 (1H, d, *J* = 2.6 Hz), 7.47 (2H, d, *J* = 7 Hz), 7.42–7.36 (3H, m), 7.31 (1H, dd, *J* = 8.7, 2.8 Hz), 7.03 (1H, d, *J* = 8.8 Hz), 4.96 (2H, s); ^13^C NMR (125 MHz, DMSO-*d*_6_) *δ* 163.4, 155.6, 135.7, 128.8, 128.29, 128.27, 126.9, 124.5, 122.7, 117.87, 115.5, 113.2, 77.0; LRMS (ES^+^), *m*/*z* 259.1 [M+H^+^]; HRMS, calcd mass for C_14_H_15_N_2_O_3_^+^ [M+H^+^]: 259.1077. Found: 259.1069 (3.3 ppm).

#### 2.4.4 General method for the synthesis of the amide series

Compound **11** (200 mg, 0.592 mmol), DMAP (cat.) and the acyl chloride (0.592 mmol) were dissolved in THF (2 mL) and DCE (0.5 mL). Pyridine (96 μL, 1.184 mmol) was added, and the reaction stirred for 24 h. The mixture was diluted with DCM (10 mL) and water (10 mL) then stirred vigorously. The mixture was passed through a phase separator, and then the solvent was removed. The crude was recrystallized from MeOH/Et_2_O. Characterisation of compounds **12**–**19** is reported in the Supplementary data.

#### 2.4.5 General method for amide deprotection

A 0.05 M solution of benzyl-protected amide (**12**–**19**) and AcOH (2 equiv) in MeOH/THF, MeOH or THF, was passed through a H_2_ flow reactor (1 mL/min, 30 °C, 1 atm) using a 20% Pd(OH)_2_ catalyst cartridge. The solvent was removed and the product was recrystallized from THF/MeOH, or washed with THF or MeOH. Characterisation of compounds **20**–**27** is reported in the Supplementary data.

## 3 Results and discussion

### 3.1 Fragment screening

**Figure 2:**
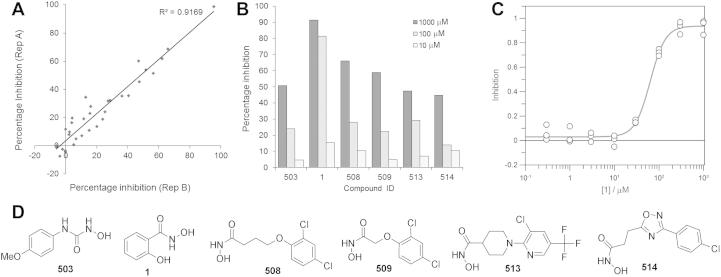
*T. brucei* GlcNAc-PI de-*N*-acetylase inhibitors. The activity of the recombinant enzyme against the synthetic substrate d-glucosamine-α-(1-6)-d-myo-inositol-1-octadecyl phosphate was measured using a mass spectrometry based assay (A) Fragments were screened at 1 mM in duplicate. (B) The six most potent fragments showed dose–response. (C) For the most potent compound **1** an eight-point potency curve was determined in triplicate giving an IC_50_ = 63 ± 4 μM. (D) Structures of the six most potent fragments.

To discover more drug-like scaffolds for zinc-binding de-*N*-acetylase inhibitors we screened a small focused library of zinc binding fragments. Because we have previously shown that the enzyme may be inhibited by substrate analogues containing hydroxamic acid, *N*-hydroxyurea or carboxylic acid groups,^12,13^ we selected 26 commercially available fragments, which contained these zinc binding moieties, with an average molecular weight of 220 (Supplemental Table S1). We screened the inhibition of recombinant *T. brucei* de-*N*-acetylase by the fragments using a mass spectrometry-based activity assay to directly monitor the conversion of the synthetic substrate *N*-acetyl-d-glucosamine-α-(1-6)-d-myo-inositol-1-octadecyl phosphate (GlcNAc-I*P*C_18_) to d-glucosamine-α-(1-6)-d-myo-inositol-1-octadecyl phosphate (GlcNAc-I*P*C_18_). The replacement of the diacylglycerol moiety of GlcNAc-PI with a simple C_18_ alkyl chain in GlcNAc-I*P*C_18_ has no effect on the substrate recognition, and enables the use of a unique mass-transition to follow the reaction.^6^ Initial screening of the fragments at 1 mM in duplicate revealed six compounds with >50% inhibition (Fig. 2A + D). However, only one compound showed >50% inhibition at 100 μM (Fig. 2B). The most potent compound **1** (Fig. 2C) had an IC_50_ of 63 ± 4 μM and a molecular weight of 153, giving an impressive ligand efficiency (-RT.lnIC_50_/N_non-H atoms_)^16^ of −0.57 kcal mol^−1^ per non-hydrogen atom, which is over one third of the theoretical maximum affinity for organic compounds of −1.5 kcal mol^−1^ per non-hydrogen atom.^17^ By way of comparison, the previously reported glucocyclitol-phospholipid substrate analogue 1R,2R-1-O-[2-C-(carboxymethyl *N*-hydroxyamide)-2-deoxy-β-d-glucopyranosyl]-cyclohexanediol 2-(*n*-octadecylphosphate) has an IC_50_ of 19 ± 0.5 μM and a molecular weight of 666.4,^13^ giving a substantially lower ligand efficiency of only −0.13 kcal mol^−1^ per non-hydrogen atom.

The result of the fragment screen compared favourably to our screening of 16,037 lead-like compounds from the Dundee Drug Discovery Unit’s compound collection^18^ against the *T. brucei* de-*N*-acetylase in a cell-based functional complementation assay,^i^ where no inhibitors were identified (data not shown). Interestingly, compound **1** (also known as SHAM) has previously been identified as an inhibitor of trypanosome alternative oxidase (TAO), and is toxic in vivo to the clinically relevant bloodstream form *T. brucei.*^19,20^

### 3.2 Inhibition of the *T. brucei* cell-free system

**Figure 3:**
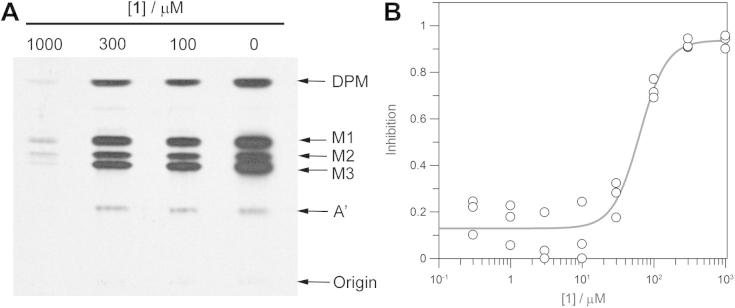
Trypanosome GPI biosynthesis in the cell-free system. (A) The *T. brucei* cell-free system was incubated with GlcNAc-PI (10 μM) in the presence of 0, 100, 300, or 1000 μM of **1** and 0.5 μCi of GDP-[^3^H]-mannose to stimulate the production of radiolabelled mannosylated GPI intermediates. Glycolipid products were extracted, separated by high-performance thin-layer chromatography, and visualised by fluorography. DPM–dolichol-phosphate-mannose, M1–Man_1_GlcN-I*P*C_18_, M2–Man_2_GlcN-I*P*C_18_, M3–Man_3_GlcN-I*P*C_18_, A′–EtN*P*Man_3_GlcN-I*P*C_18_. (B) Inhibition of the turnover of GlcNAc-I*P*C_18_ (10 μM) by the presence of **1** measured in the *T. brucei* cell-free system in the LC–MS/MS assay.

The ability of **1** to act as an inhibitor of the *T. brucei* GPI pathway was confirmed using the trypanosome cell-free system (Fig. 3). Priming the cell-free system with GlcAc-I*P*C_18_ in the presence of GDP-[^3^H]-mannose stimulates the production of radiolabelled mannosylated GPI intermediates that can be separated by high performance thin-layer chromatography and visualised by fluorography. Priming the cell-free system with GlcNAc-I*P*C_18_ (10 μM) was prevented by incubation with >300 μM of **1** (Fig. 3B), a five-fold increase over the IC_50_ value against the recombinant enzyme. To verify that the decrease in potency was not due to a reduction in potency against the endogenous enzyme compared with the recombinant enzyme, we repeated the mass spectrometry based activity assay with the *T. brucei* cell-free system. We found that it was necessary to reduce the amount of cell-free system by 40-fold in the mass spectrometry-based assay compared with the radiometric assay to achieve measurements in the linear range for the turnover of GlcNAc-I*P*C_18_. The potency of compound **1** against the cell-free system with an IC_50_ = 66 ± 8 μM (Fig. 3B) was comparable to that against the recombinant enzyme.

### 3.3 Structure–activity relationship

Substructure searching identified commercially available analogues of **1** that were screened for activity against in the mass spectrometry based assay against *T. brucei* cell-free system. Replacement of the hydroxamic acid with carboxylic acid decreased the potency of inhibition, potentially due to the carboxylic acid acting as a less efficient zinc chelator. Removal of the 2-hydroxyl group or substitution with bromide or amine completely abrogated the inhibitory activity, whereas substitution at the 4′ position with bromine was tolerated (Table 1).

**Scheme 1:**
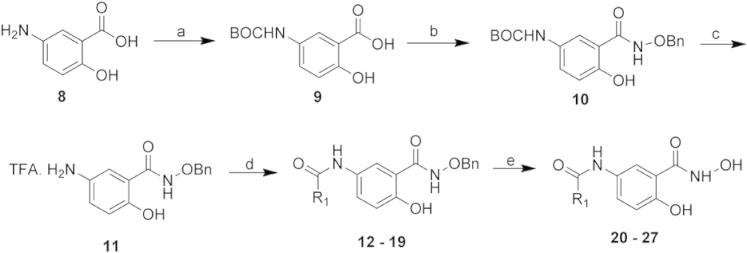
Synthesis of a small array of 5′ amide derivatives of **1**. Reagents and conditions: (a) TEA, Boc_2_O, dioxane, RT, 18 h, 76%. (b) BnONH_2_·HCl, DCC, DMAP, TEA, CHCl_3_/THF, 0 °C, 16 h to RT, 24 h, 46%. (c) TFA (wet), RT, 1 h, 85%. (d) R_1_-COCl, pyridine, DMAP (cat.), THF/DCE 4:1, RT, 24 h, 21–79%. (e) AcOH, H_2_, 20% Pd(OH)_2_, MeOH/THF, 0.5 mL/min, 1 atm, 45–100%.

To further explore the available chemical space, a small array of analogues of compound **1** were synthesised from the literature compound **11**^21^ by coupling to the 5-NH_2_ position with commercially available acid chlorides (Scheme 1). After deprotection of the hydroxamic acid, the majority of compounds showed decreased inhibitory activity compared with **1** (Table 2). The two most potent compounds **20** and **21** had IC_50_ values of 45 ± 11 μM and 69 ± 6 μM respectively (Supplementary Fig. 1), comparable to the parent compound albeit with reduced ligand efficiency (−0.30 and −0.29 kcal mol^−1^ per non-hydrogen atom respectively), suggesting that the 5′-amide substitution was tolerated but contributed little to binding.

**Figure 4:**
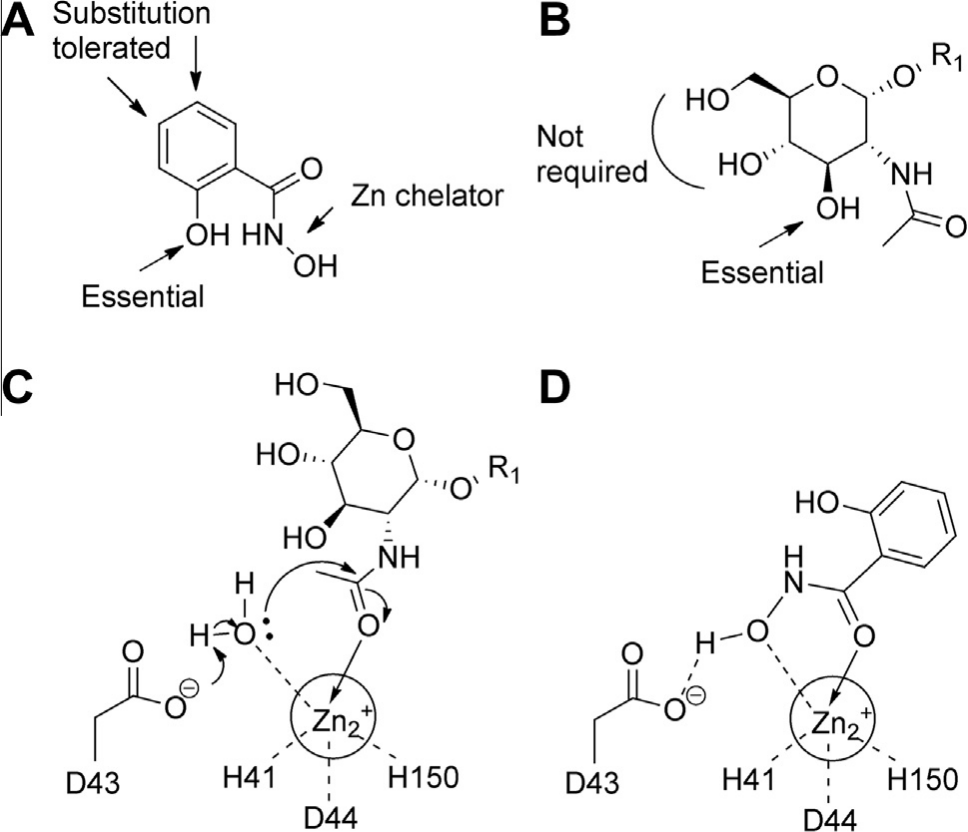
Proposed mode of action. (A) Structure–activity relationships of **1**.(B) Components of the natural substrate GlcNAc-PI recognised by the enzme. R_1_ = α(1-6)-d-myo-inositol-1-phosphate-dimyristolglycerol. (C) The GlcNAc-PI de-*N*-acetylase contains catalytic zinc chelated by the residues H49, D52 and H157, and the catalytic base D43. (D) Compound **1** could inhibit the enzyme through binding of the hydroxamic acid to the catalytic zinc, replacing both the acetamidocarbonyl and activated water.

The structure–activity relationship derived from the data presented here suggest that the 2-OH group of **1** is essential for activity and substitution at the 4- and 5- position is tolerated (Fig. 4). Comparing **1** with the natural substrate GlcNAc-PI could suggest that **1** is mimicking the GlcNAc moiety, with the 2-OH of **1** mimicking the 3′-OH that is essential for substrate recognition. In the proposed reaction mechanism for the de-*N*-acetylase, the catalytic base D43 assists the nucleophilic attack of a zinc-bound water molecule on the activated acetamidocarbonyl, and the subsequent protonation of the tetrahedral intermediate promotes loss of acetic acid. We propose that **1** could inhibit the enzyme through the hydroxamic acid binding to the catalytic zinc, and replacing both the acetamidocarbonyl and the activated water (Fig. 4).

### 3.4 Conclusions

We have identified salicylic hydroxamic acid **1** as an inhibitor of *T. brucei* GlcNAc-PI de-*N*-acetylase with high ligand efficiency, representing the first non-substrate analogue inhibitor of the trypanosome GPI pathway. Although **1** shows modest potency with an IC_50_ of 63 ± 4 μM, it has impressive ligand efficiency of 0.57 kcal mol^−1^ per non-hydrogen atom, and thus may be a useful starting point to develop more potent inhibitors.

## Figures and Tables

**Table 1:** Inhibitory activity of commercially available analogues of **1**

**Figure:**
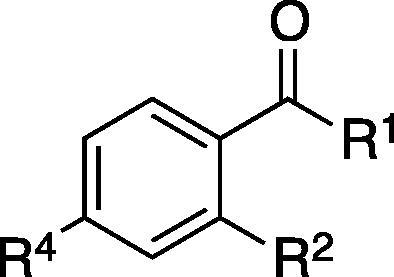

ID	R^1^	R^2^	R^4^	Inhibition at 1 mM^a^ (%)
**1**	NHOH	OH	H	97 ± 2
**2**	OH	OH	H	25 ± 1
**3**	NHOH	H	H	−6 ± 14
**4**	NHOH	Br	H	−4 ± 15
**5**	NHOH	NH_2_	H	−5 ± 2
**6**	NHOH	OH	Br	86 ± 2
**7**	NHOH	H	Br	9.6 ± 15

a Inhibition of *T. brucei* de-*N*-acetylase (cell-free system).

**Table 2:** Inhibitory activity of novel derivatives of **1**

ID	R_1_	Inhibition at 1 mM^a^ (%)
**20**		99 ± 1.6
**21**		96 ± 0.4
**22**		62 ± 24
**23**		25 ± 14
**24**		40 ± 17
**25**		75 ± 7.0
**26**		56 ± 8.3
**27**		86 ± 11

a Inhibition of *T. brucei* de-*N*-acetylase (cell-free system).

## Supplementary data

Supplementary data This document contains supplementary figure, table and spectral data.

## Footnotes

i The assay used the CHO MS2S cell line, which has a defective de-*N*-acetylase and expresses the GPI anchored human CD55, functionally complemented by transfection with *T. brucei* de-*N*-acetylase. Cells were treated with trypsin, plated onto compound, and GPI biosynthesis monitored by αCD55 ELISA at 48 h.
