# Supplementary material for: Fragment screening reveals salicylic hydroxamic acid as an inhibitor of Trypanosoma brucei GPI GlcNAc-PI de-N-acetylase
Source: Carbohydr Res. 2014 Mar 31;387(100):54–8. doi: 10.1016/j.carres.2013.12.016 (PMC3991331; doi:10.1016/j.carres.2013.12.016)
Supplement: Supplementary data — This document contains supplementary figure, table and spectral data. [file mmc1.docx]

Fragment screening reveals salicylic hydroxamic acid as an inhibitor of *Trypanosoma brucei* GPI GlcNAc-PI de-*N*-acetylase.

Michael D. Urbaniak,^§1^ Amy Capes, ^§^ Arthur Crossman, Sandra O’Neill, Stephen Thompson, Ian Gilbert, Michael A. J. Ferguson*

Division of Biological Chemistry and Drug Discovery, College of Life Sciences, University of Dundee, Dow Street, Dundee, DD1 5EH, UK.

* Corresponding author. Phone +44 (0) 1382 384219, Email [m.a.j.ferguson@dundee.ac.uk](mailto:m.a.j.ferguson@dundee.ac.uk)

^§^ These authors contributed equally to the work.

^1^ Present Address: Division of Biomedical and Life Sciences, Faculty of Health and Medicine, Lancaster University, Lancaster, LA1 4GQ, UK.

**Supplementary data:**

**Compound characterisation (12–27).**

**Table S1**. Fragment library screening data.

**Figure S1**. IC_50_ curves of compounds **20** and **21**.

**Compound characterisation**

**N-(Benzyloxy)-5-(cyclohexanecarboxamido)-2-hydroxybenzamide (12)**

White powder (99 mg, 45%), mp 186 °C. ^1^H NMR (500 MHz, DMSO-d_6_) *δ* 11.44 (1H, bs), 11.05 (1H, bs), 9.54 (1H, bs), 7.91 (1H, d, *J* = 2.5 Hz), 7.52 (1H, dd, *J* = 8.8, 2.5 Hz), 7.47 (2H, d, *J* = 6.8 Hz), 7.43-7.35 (3H, m), 6.86 (1H, d, *J* = 8.9 Hz), 4.96 (2H,s ), 2.30 (1H, tt, *J* = 11.4, 3.3 Hz), 1.81-1.76 (4H, m), 1.66 (1H, d, *J* = 11.25 Hz), 1.48-1.38 (2H, m), 1.32-1.17 (3H, m); ^13^C NMR (125 MHz, DMSO-d_6_) *δ* 173.8, 165.2, 153.3, 135.8, 131.08, 128.8, 128.3, 125.3, 119.8, 116.8, 115.6, 77.1, 44.6, 29.1, 25.4, 25.2; LRMS (ES^+^), *m/z* 369.2 [M+H^+^]; HRMS, calcd. mass for C_21_H_25_N_2_O_4_^+^ [M+H^+^]: 369.1809. Found: 369.1798 (2.8 ppm).

**5-Benzamido-N-(benzyloxy)-2-hydroxybenzamide (13)**

Off-white powder (116 mg, 54%), mp 190 °C. ^1^H NMR (500 MHz, DMSO-d_6_) *δ* 11.54 (1H, s), 11.26 (1H, s), 10.19 (1H, s), 8.08 (1H, d, *J* = 2.5 Hz), 7.97 (2H, d, *J* = 7.1 Hz), 7.71 (1H, dd, *J* = 8.9, 2.7 Hz), 7.59 (1H, t, *J* = 7.4 Hz), 7.53 (2H, t, *J* = 7.7 Hz), 7.49 (2H, d, *J* = 7.1 Hz), 7.43-7.36 (3H, m), 6.94 (1H, d, *J* = 8.9 Hz), 4.96 (2H, s,); ^13^C NMR (125 MHz, DMSO-d_6_) *δ* 165.1, 153.9, 135.8, 134.7, 131.5, 130.6, 129.2, 128.8, 128.5, 128.4, 128.3, 127.5, 126.7, 121.3, 116.8, 115.7, 77.2; LRMS (ES^+^), *m/z* 363.1 [M+H^+^]; HRMS, calcd. mass for C_21_H_19_N_2_O_4_^+^ [M+H^+^]: 363.1339. Found: 363.1324 (4.1 ppm).

**N-(Benzyloxy)-2-hydroxy-5-(4-methylbenzamido)benzamide (14)**

White powder (110 mg, 49%), mp 154-155 °C. ^1^H NMR (500 MHz, DMSO-d­_6_) *δ* 11.47 (1H, s), 11.11 (1H, s), 10.53 (1H, s), 8.07 (1H, s), 7.64 (1H, d, *J* = 7.4 Hz), 7.49-7.28 (7H, m), 7.30 (2H, d, *J* = 9.1 Hz), 6.92 (1H, d, *J* = 8.8 Hz), 4.97 (2H, s), 2.41 (3H, s); ^13^C NMR (125 MHz, DMSO-d­_6_) *δ* 167.5, 165.1, 153.7, 137.1, 135.8, 135.2, 130.8, 130.5, 129.5, 128.9, 128.3, 127.1, 125.6, 120.5, 116.9, 115.9, 77.1, 19.3; LRMS (ES^+^), *m/z* 377.1 [M+H^+^]; HRMS, calcd. mass for C_22_H_21_N_2_O_4_^+^ [M+H^+^]: 377.1496. Found: 377.1496 (0 ppm).

**N-(Benzyloxy)-2-hydroxy-5-stearamidobenzamide (15)**

Pale pink powder (246 mg, 79%), mp 85-87 °C. ^1^H NMR (500 MHz, DMSO-d­_6_) *δ* 11.49 (1H, s), 11.12 (1H, s), 9.74 (1H, s), 7.88 (1H, s), 7.54 (1H, d, *J* = 8.5 Hz), 7.55-7.37 (5H, m), 6.86 (1H, d, *J* = 8.7 Hz), 4.94 (2H, s), 2.25 (2H, t, *J* = 6.1 Hz), 1.58 (2H, bs), 1.24 (28H, s), 0.86 (3H, t, *J* = 6.1 Hz); ^13^C NMR (125 MHz, DMSO-d­_6_) *δ* 170.8, 165.2, 153.3, 135.8, 131.0, 128.8, 128.6, 128.3, 128.2, 125.2, 119.8, 116.8, 115.7, 77.1, 36.1, 31.3, 29.0, 29.0, 28.9, 28.83, 28.78, 28.71, 28.67, 28.65, 25.1, 22.1, 13.9; LRMS (ES^+^), *m/z* 525.4 [M+H^+^]; HRMS, calcd. mass for C_32_H_49_N_2_O_4_^+^ [M+H^+^]: 525.3687. Found: 525.3674 (2.4 ppm).

**1-Acetyl-N-(3-((benzyloxy)carbamoyl)-4-hydroxyphenyl)piperidine-4-carboxamide (16)**

White powder (52 mg, 21%), mp 195 °C. ^1^H NMR (500 MHz, DMSO-d­_6_) *δ* 11.43 (1H, bs), 11.06 (1H, bs), 9.68 (1H, bs), 7.90 (1H, d, *J* = 2.5 Hz), 7.53 (1H, dd, *J* = 8.8, 2.5 Hz), 7.47 (2H, d, *J* = 6.8 Hz), 7.42-7.35 (3H, m), 6.87 (1H, d, *J* = 8.8 Hz), 4.96 (2H, s), 4.39 (1H, d, *J* = 12.0 Hz), 3.87 (1H, d, *J* = 12.6 Hz), 3.09 (1H, t, *J* = 11.1 Hz), 2.63 (1H, t, *J* = 11.1 Hz), 2.59-2.53 (1H, m), 2.02 (3H, s), 1.81 (2H, t, *J* = 11.9 Hz), 1.61 (1H, q, *J* = 10.0 Hz), 1.48 (1H, q, *J* = 10.8 Hz); ^13^C NMR (125 MHz, DMSO-d­_6_) *δ* 172.5, 168.0, 165.1, 153.4, 135.8, 130.9, 128.8, 128.3, 125.3, 119.9, 116.9, 115.7, 77.1, 45.2, 42.4, 40.8, 28.7, 28.1, 21.3; LRMS (ES^+^), *m/z* 412.2 [M+H^+^]; HRMS, calcd. mass for C_22_H_26_N_3_O_5_^+^ [M+H^+^]: 412.1867. Found: 412.1871 (-0.9 ppm).

**N-(3-((Benzyloxy)carbamoyl)-4-hydroxyphenyl)-[1,1'-biphenyl]-4-carboxamide (17)**

Pale pink powder (65 mg, 25%), mp 251-252 °C. ^1^H NMR (500 MHz, DMSO-d­_6_) *δ* 11.49 (1H, s), 11.18 (1H, s), 10.15 (1H, s), 8.10 (1H, d, *J* = 2.7 Hz), 8.08 (2H, d, *J* = 8.4 Hz), 7.83 (2H, d, *J* = 8.4 Hz), 7.77 (2H, d, *J* = 7.3 Hz), 7.73 (1H, dd, *J* = 8.9, 2.7 Hz), 7.54-7.36 (8H, m), 6.95 (1H, d, *J* = 8.8 Hz), 4.99 (2H, s); ^13^C NMR (125 MHz, DMSO-d­_6_) *δ* 165.1, 164.7, 153.9, 143.0, 139.1, 135.8, 133.4, 130.7, 129.9, 129.3, 128.8, 128.3, 128.2, 128.1, 126.93, 126.88, 126.8, 126.7, 126.6, 121.4, 116.8, 115.7, 77.2; LRMS (ES^+^), *m/z* 439.2 [M+H^+^]; HRMS, calcd. mass for C_27_H_23_N_2_O_4_^+^ [M+H^+^]: 439.1652. Found: 439.1631 (4.8 ppm).

**N-(3-((Benzyloxy)carbamoyl)-4-hydroxyphenyl)tetrahydro-2H-pyran-4-carboxamide (18)**

Pale grey-pink powder (129 mg, 59%), mp 144-145 °C. ^1^H NMR (500 MHz, DMSO-d­_6_) *δ* 11.44 (1H, bs), 11.0741 (1H, bs), 9.64 (1H, s), 7.91 (1H, d, *J* = 2.6 Hz), 7.53 (1H, dd, *J* = 8.8, 2.4 Hz), 7.47 (2H, d, *J* = 6.8 Hz), 7.43-7.35 (3H, m), 6.87 (1H, d, *J* = 8.8 Hz), 4.96 (2H, s), 3.92 (2H, dt, *J* = 11.1, 3.2 Hz), 3.42-3.33 (2H, m), 2.60-2.53 (m, 1H, CH, H13), 1.72-1.65 (m, 4H, CH_2_, H14, H17); ^13^C NMR (125 MHz, DMSO-d­_6_) *δ* 171.5, 165.2, 153.4, 135.8, 130.9, 128.8, 128.3, 125.4, 119.9, 116.9, 115.6, 77.1, 66.4, 41.5, 28.9; LRMS (ES^+^), *m/z* 371.1 [M+H^+^]; HRMS, calcd. mass for C_20_H_23_N_2_O_5_^+^ [M+H^+^]: 371.1601. Found: 371.1619 (-4.8 ppm).

**N-(3-((Benzyloxy)carbamoyl)-4-hydroxyphenyl)benzo[d][1,3]dioxole-5-carboxamide (19)**

Pale gray-pink powder (127 mg, 53%), mp 221 °C. ^1^H NMR (500 MHz, DMSO-d­_6_) *δ* 11.54 (1H, s), 11.25 (1H, bs), 10.01 (1H, s), 8.04 (1H, d, *J* = 2.6 Hz), 7.68 (1H, dd, *J* = 8.9, 2.6 Hz), 7.59 (1H, dd, *J* = 8.2, 1.8 Hz), 7.52 (1H, d, *J* = 1.8 Hz), 7.48 (2H, d, *J* = 7.0 Hz), 7.43-7.36 (3H, m), 7.06 (1H, d, *J* = 8.2 Hz), 6.92 (1H, d, *J* = 8.8 Hz), 6.14 (2H, s), 4.96 (2H, s); ^13^C NMR (125 MHz, DMSO-d­_6_) *δ* 165.2, 164.1, 153.9, 150.0, 147.4, 135.8, 130.7, 128.8, 128.5, 128.3, 126.7, 122.7, 121.4, 116.8, 115.6, 107.9, 107.5, 101.8, 77.2; LRMS (ES^+^), *m/z* 407.1 [M+H^+^]; HRMS, calcd. mass for C_22_H_19_N_2_O_6_^+^ [M+H^+^]: 407.1238. Found: 407.1237 (0.1 ppm).

**5-(Cyclohexanecarboxamido)-N,2-dihydroxybenzamide (20)**

White powder (37 mg, 52%), mp 229-230 °C. ^1^H NMR (500 MHz, MeOD) *δ* 7.86 (1H, d, *J* = 2.4 Hz), 7.48 (1H, dd, *J* = 8.9, 2.5 Hz), 6.90 (1H, d, Ar *J* = 8.9 Hz), 2.35 (1H, tt, *J* = 11.8, 3.4 Hz), 1.87 (4H, t, *J* = 14.8 Hz), 1.74 (1H, d, *J* = 12.2 Hz), 1.54 (2H, ddd, *J* = 24.6, 12.75, 2.9 Hz), 1.42-1.28 (3H, m); ^13^C NMR (125 MHz, MeOD) *δ* 177.6, 156.5, 131.6, 127.8, 121.4, 118.3, 115.8, 47.0, 30.7, 26.9, 26.8; LRMS (ES^+^), *m/z* 279.1 [M+H^+^]; HRMS: calcd. mass for C_14_H_19_N_2_O_4_^+^ [M+H^+^]: 279.1339. Found: 279.1345 (-2.2 ppm).

**5-Benzamido-N,2-dihydroxybenzamide (21)**

White powder (48 mg, 57%), mp 211 °C. ^1^H NMR (500 MHz, MeOD) *δ* 7.87 (1H, d, *J =* 2.4 Hz), 7.81 (2H, d, *J* = 7.2 Hz), 7.51 (1H, dd, *J* = 8.9, 2.5 Hz), 7.46 (1H, tt, *J* = 7.4, 1.15 Hz), 7.38 (2H, t, *J* = 7.8 Hz), 6.82 (1H, d, *J* = 8.8 Hz); ^13^C NMR (125 MHz, MeOD) *δ* 168.9, 157.0, 136.0, 133.0, 131.4, 129.7, 128.8, 128.6, 122.5, 118.4, 115.9; LRMS (ES^+^), *m/z* 273.1 [M+H^+^]; HRMS, calcd. mass for C_14_H_13_N_2_O_4_^+^ [M+H^+^], 273.0870. Found: 273.0874 (-1.6 ppm).

**N,2-Dihydroxy-5-(4-methylbenzamido)benzamide (22)**

White powder (33 mg, 45%), mp 209 °C. ^1^H NMR (500 MHz, MeOD) *δ* 8.01 (1H, d, *J* = 2.5 Hz), 7.63 (1H, dd, *J* = 8.9, 2.5 Hz), 7.48 (1H, d, *J* = 7.6 Hz), 7.39 (1H, dd, *J* = 7.6, 1.2 Hz), 3.32-7.30 (2H, m), 6.95 (1H, d, *J* = 8.8 Hz), 2.48 (3H, s); ^13^C NMR (125 MHz, MeOD) *δ* 171.3, 156.9, 138.1, 137.0, 131.9, 131.5, 131.1, 128.1, 126.9, 121.6, 118.6, 118.5, 115.9, 19.7; LRMS (ES^+^), *m/z* 287.1 [M+H^+^]; HRMS, calcd. mass for C_15_H_15_N_2_O_4_^+^ [M+H^+^]: 287.1026. Found: 287.1023 (1.2 ppm).

**N,2-Dihydroxy-5-stearamidobenzamide (23)**

White powder (153 mg, 80%), mp 185-187 °C. ^1^H NMR (500 MHz, THF-d_8_, 50 °C) *δ* 11.51 (1H, bs), 10.80 (1H, bs), 8.56 (1H, s), 8.31 (1H, bs), 7.97 (1H, s), 7.28 (1H, s), 6.77 (1H, d, *J* = 8.8 Hz), 2.24 (2H, t, *J* = 7.3 Hz), 1.68-1.66 (2H, m), 1.33-1.26 (28H, m), 0.89 (3H, t, *J* = 7.0 Hz); ^13^C NMR (125 MHz, THF-d­_8_, 50 °C) *δ* 170.1, 156.7, 130.9, 125.2, 117.3, 117.2, 112.9, 36.6, 31.8, 29.6, 29.4, 29.3, 29.2, 22.5, 13.3; LRMS (ES^+^), *m/z* 435.3 [M+H^+^]; HRMS, calcd. mass for C_25_H_43_N_2_O_4_^+^ [M+H^+^]: 435.3217. Found: 435.3215 (0.5 ppm).

**1-Acetyl-N-(4-hydroxy-3-(hydroxycarbamoyl)phenyl)piperidine-4-carboxamide (24)**

White powder (24 mg, 65%), mp 211-212 °C. ^1^H NMR (500 MHz, MeOD) *δ* 7.89 (1H, d, *J* = 2.1 Hz), 7.49 (1H, dd, *J* = 8.9, 2.4 Hz), 6.90 (1H, d, *J* = 8.8 Hz), 4.58 (1H, d, *J* = 13.3 Hz), 4.03 (1H, d, *J* = 13.8 Hz), 3.21 (1H, dd, *J* = 25.9, 2.4 Hz), 2.75 (1H, dd, *J* = 25.8, 2.5 Hz), 2.66-2.61 (1H, m), 2.14 (3H, s), 1.92 (2H, t, *J* = 13.9 Hz), 1.77 (1H, ddd, *J* = 24.5, 12.5, 4.25 Hz), 1.66 (1H, ddd, *J* = 24.6, 12.6, 4.3 Hz); ^13^C NMR (125 MHz, MeOD) *δ* 175.5, 171.6, 156.6, 131.5, 127.7, 121.5, 118.4, 115.9, 47.1, 44.4, 42.3, 30.1, 29.6, 21.2; LRMS (ES^+^), *m/z* 322.1 [M+H^+^]; HRMS, calcd. mass for C_15_H_20_N_3_O_5_^+^ [M+H^+^]: 322.1397. Found: 322.1383 (4.5 ppm).

**N-(4-Hydroxy-3-(hydroxycarbamoyl)phenyl)-[1,1'-biphenyl]-4-carboxamide (25)**

White powder (33 mg, 69%), mp 216 °C. ^1^H NMR (500 MHz, MeOD/THF-d_8_) *δ* 8.03 (1H, s), 8.01 (2H, d, *J* = 8.2 Hz), 7.74 (2H, d, *J* = 8.2 Hz), 7.65 (2H, d, *J* = 7.4 Hz), 7.59 (1H, dd, *J* = 8.8, 1.5 Hz), 7.43 (2H, t, *J* = 7.5 Hz), 7.34 (1H, t, *J* = 7.4 Hz), 6.87 (1H, d, *J* = 8.9 Hz); ^13^C NMR (125 MHz, MeOD/THF-d_8_) *δ* 167.9, 157.4, 145.8, 141.4, 135.0, 131.7, 130.1, 129.3, 129.2, 128.5, 128.2, 128.1, 121.7, 118.6, 115.6, 109.0; LRMS (ES^+^), *m/z* 349.1091 [M+H^+^]; HRMS, calcd. mass for C_20_H_17_N_2_O_4_^+^ [M+H^+^]: 349.1184. Found: 384.1180 (0.8 ppm).

**N-(4-Hydroxy-3-(hydroxycarbamoyl)phenyl)tetrahydro-2H-pyran-4-carboxamide (26)**

White powder (94 mg, 100%), mp 209 °C. ^1^H NMR (500 MHz, MeOD/THF-d_8_) *δ* 7.83 (1H, d, *J* = 2.6 Hz), 7.38 (1H, d, *J* = 8.8 Hz), 6.78 (1H, dd, *J* = 8.8, 2.7 Hz), 3.91-3.89 (2H, m), 3.39-3.35 (2H, m), 2.52-2.38 (1H, m), 1.80-1.65 (4H, m); ^13^C NMR (125 MHz, MeOD/THF-d_8_) *δ* 174.5, 157.0, 131.4, 127.0, 119.9, 118.2, 114.8, 108.6, 67.9, 43.5, 30.2, 30.0; LRMS (ES^+^), *m/z* 281.1 [M+H^+^]; HRMS, calcd. mass for C_13_H_17_N_2_O_5_^+^ [M+H^+^]: 281.1132. Found: 281.1124 (2.7 ppm).

**N-(4-Hydroxy-3-(hydroxycarbamoyl)phenyl)benzo[d][1,3]dioxole-5-carboxamide (27)**

White powder (17 mg, 20%), mp 209 °C. ^1^H NMR (500 MHz, MeOD/THF-d_8_) *δ* 7.95 (1H, s), 7.46-7.43 (2H, m), 7.39 (1H, s), 6.87 (1H, d, *J* = 8.0 Hz), 6.84 (1H, d, *J* = 8.8 Hz), 6.01 (2H, s); ^13^C NMR (125 MHz, MeOD/THF-d_8_) *δ* 166.8, 157.7, 152.1, 149.5, 131.7, 130.3, 128.2, 123.7, 121.0, 118.6, 115.2, 109.0, 108.9, 103.4; LRMS (ES^+^), *m/z* 317.1 [M+H^+^]; HRMS, calcd. mass for C_15_H_13_N_2_O_6_^+^ [M+H^+^]: 317.0768. Found: 317.0768 (-0.1 ppm).

**Table S1.** Fragment library screening data.

| **Structure** | **ID** | **Molecular Weight** | Inhibition at 1 mM^a^ (%) |
| --- | --- | --- | --- |
|  | 501 | 167.16 | 12 ± 11 |
|  | 502 | 264.32 | 24 ± 15 |
|  | 503 | 182.18 | 53 ± 2 |
|  | 504 | 181.2 | 36 ± 0.7 |
|  | **1** | 153.14 | 97 ± 2 |
|  | 506 | 204.23 | 30 ± 3 |
|  | 507 | 180.20 | 10 ± 9 |
|  | 508 | 264.1 | 67 ± 20 |
|  | 509 | 236.1 | 62 ± 0.3 |
|  | 510 | 261.3 | 30 ± 3 |
|  | 511 | 278.3 | 17 ± 5 |
|  | 512 | 324.4 | 46 ± 3 |
|  | 513 | 323.7 | 54 ± 4 |
|  | 514 | 267.7 | 54 ± 9 |
|  | 515 | 210.3 | 22 ± 9 |
|  | 516 | 206.2 | 19 ± 5 |
|  | 517 | 196.2 | 6 ± 5 |
|  | 518 | 210.2 | 6 ± 8 |
|  | 519 | 186.2 | 21 ± 3 |
|  | **2** | 138.12 | 25 ± 1 |
|  | 521 | 249.09 | 38 ± 4 |
|  | 522 | 221.04 | 15 ± 6 |
|  | 523 | 308.7 | 9 ± 2 |
|  | 524 | 252.7 | 13 ± 3 |
|  | 525 | 123.1 | -3 ± 4 |
|  | 526 | 108.1 | -2 ± 3 |

^a^ Inhibition of *T. brucei* de-*N*-acetylase (cell-free system).


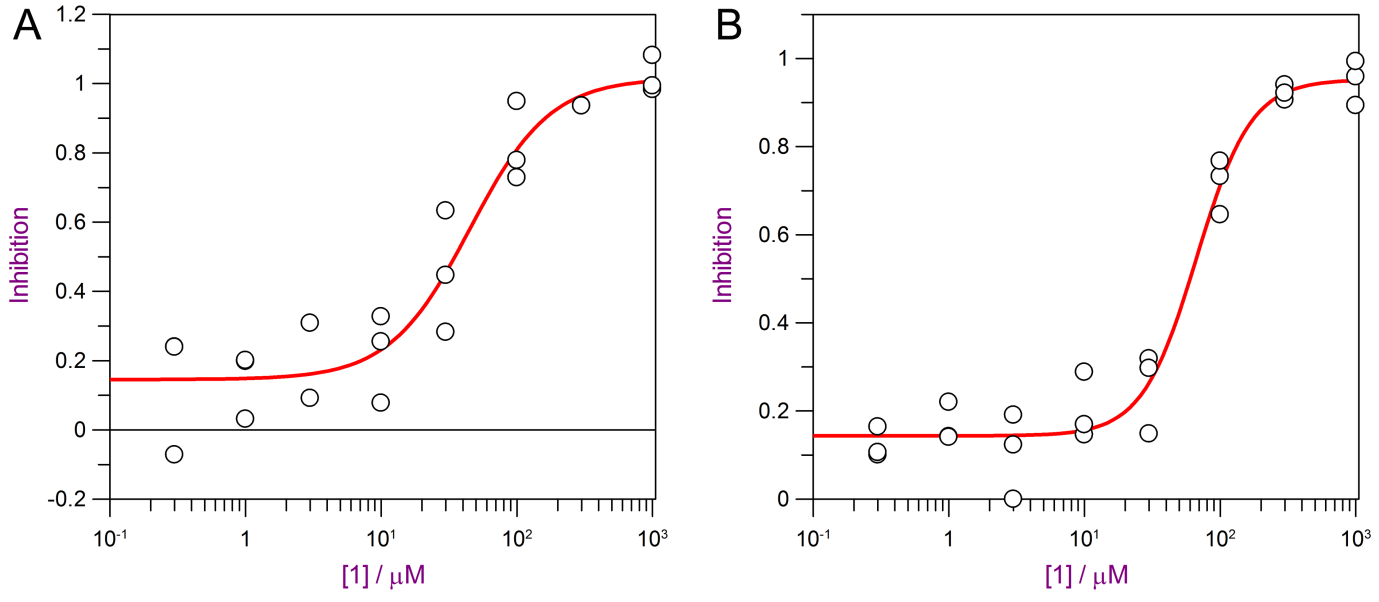


**Figure S1**. IC_50_ curves of compounds **20** and **21**. Inhibition of the turnover of GlcNAc-I*P*C_18_ (10 μM) by the *T. brucei* cell-free system in the LC-MS/MS assay. **A.** Compound **20** IC_50_ = 45 ± 11 μM. **B.** IC_50_ = 69 ± 6 μM.
